# Calpain-5 gene variants are associated with diastolic blood pressure and cholesterol levels

**DOI:** 10.1186/1471-2350-8-1

**Published:** 2007-01-16

**Authors:** María E Sáez, María T Martínez-Larrad, Reposo Ramírez-Lorca, José L González-Sánchez, Carina Zabena, María J Martinez-Calatrava, Alejandro González, Francisco J Morón, Agustín Ruiz, Manuel Serrano-Ríos

**Affiliations:** 1 Departamento de Genómica Estructural. Neocodex. Sevilla, Spain; 2 Departamento de Medicina Interna II. Hospital Clínico San Carlos. Madrid, Spain; 3 Unidad de reproducción y genética humana. Centro Avanzado de Fertilidad (CAF). Jerez de la Frontera, Cádiz, Spain

## Abstract

**Background:**

Genes implicated in common complex disorders such as obesity, type 2 diabetes mellitus (T2DM) or cardiovascular diseases are not disease specific, since clinically related disorders also share genetic components. Cysteine protease Calpain 10 (CAPN10) has been associated with T2DM, hypertension, hypercholesterolemia, increased body mass index (BMI) and polycystic ovary syndrome (PCOS), a reproductive disorder of women in which isunlin resistance seems to play a pathogenic role. The calpain 5 gene (*CAPN5*) encodes a protein homologue of CAPN10. *CAPN5 *has been previously associated with PCOS by our group. In this new study, we have analysed the association of four *CAPN5 *gene variants(rs948976A>G, rs4945140G>A, rs2233546C>T and rs2233549G>A) with several cardiovascular risk factors related to metabolic syndrome in general population.

**Methods:**

Anthropometric measurements, blood pressure, insulin, glucose and lipid profiles were determined in 606 individuals randomly chosen from a cross-sectional population-based epidemiological survey in the province of Segovia in Central Spain (Castille), recruited to investigate the prevalence of anthropometric and physiological parameters related to obesity and other components of the metabolic syndrome. Genotypes at the four polymorphic *loci *in *CAPN5 *gene were detected by polymerase chain reaction (PCR).

**Results:**

Genotype association analysis was significant for BMI (p ≤ 0.041), diastolic blood pressure (p = 0.015) and HDL-cholesterol levels (p = 0.025). Different *CAPN5 *haplotypes were also associated with diastolic blood pressure (DBP) (0.0005 ≤ p ≤ 0.006) and total cholesterol levels (0.001 ≤ p ≤ 0.029). In addition, the AACA haplotype, over-represented in obese individuals, is also more frequent in individuals with metabolic syndrome defined by ATPIII criteria (p = 0.029).

**Conclusion:**

As its homologue *CAPN10*, *CAPN5 *seems to influence traits related to increased risk for cardiovascular diseases. Our results also may suggest *CAPN5 *as a candidate gene for metabolic syndrome.

## Background

Factors that increase the risk of cardiovascular disease (CVD) include obesity, dyslipidemia, glucose intolerance, type 2 diabetes mellitus (T2DM) and hypertension. When these factors cluster in an individual is called metabolic syndrome (MS), a complex disorder characterized by impaired glucose metabolism, dyslipidemia, hypertension and obesity [1].

Calpains are a class of cysteine proteases that requires calcium for its activation. They are implicated in a wide range of cellular functions, including apoptosis, intracellular signalling, proliferation and differentiation [2]. The human calpain family currently comprises 16 members and two subfamilies based on its functional domains [3]. The classic calpains are heterodimeric proteins that contain five sets of EF-hand calcium-binding structures in each subunit, similar to those in calmodulin. Calpain 10 (CAPN10), calpain 5 (CAPN5) and calpain 6 (CAPN6) proteins belong to the non-EF-hand subfamily that lacks these calcium-binding motifs; these calpains also differ from the remaining members of this subfamily in the presence of a T-domain homologous to *C.elegans *TRA-3, a protein implicated in the sexual determination of the hermaphrodite worm [4,5]. In fact, calpain 5 is the human ortologue of the TRA-3 nematode protein, a protease that exerts its action over a transmembrane protein called TRA-2 which binds and inhibits the male promoting FEM proteins (FEM-1, FEM-2 and FEM-3) [6,7]. Interestingly, the knockout mice of one of the three mammalian homologues of the *C.elegans *fem-1 gene, Fem1b, display abnormal glucose homeostasis, with abnormal glucose tolerance tests and defective glucose-stimulated insulin secretion [8]. In humans, the *FEM1A *gene maps to chromosome 19p13.3, a region linked to polycystic ovary syndrome (PCOS), a common endocrine disorder of women of reproductive age, characterized by chronic anovulation, infertility, hyperandrogenemia and frequently, insulin resistance resulting in an increased prevalence of obesity, CVD and T2DM, reason why PCOS is consider a phenotype closely related to metabolic syndrome. Maher y cols. [9] have recently reported a germline missense mutation in the *FEM1A *gene in a PCOS woman that was absent in 198 control chromosomes; the authors propose *FEM1A *as a candidate gene for PCOS.

The *CAPN5 *homologue *CAPN10 *was identified as a T2DM susceptibility *locus *by Horikawa *et al*. [10] and has been shown to be related to proinsulin processing, insulin secretion and insulin resistance [11,12]. Ehrman et al. [13] and our group [14] independently published association of *CAPN10 *gene with PCOS. A reanalysis of *CAPN10 *gene in a larger PCOS population, allowed us to identify specific haplotypes associated with hypercholesterolemia in PCOS patients [15]. Furthermore, *CAPN10 *has been associated with hypertension and elevated body mass index (BMI) by different groups [16,17].

In a previous work, we analyzed four *CAPN5 *gene variants (rs948976, rs4945140, rs2233546 and rs2233549) in 148 PCOS women [18]. We found that specific *CAPN5 *haplotypes were overrepresented in PCOS patients. In addition, we identified several *CAPN5 *alleles associated with phenotypic differences observed between PCOS patients, such as the presence of obesity, cardiovascular complications, and familial antecedents of obesity, hypertension and T2DM aggregation. These findings aimed us to investigate the relation of *CAPN5 *gene with cardiovascular risk factors in the general population. Silander *et al*. [19] have reported strong evidence of linkage within 11q14, the chromosomal region that contains *CAPN5 *gene in a large set of Finnish affected sibling pair families with T2DM.

Here we present the first population-based association analysis of *CAPN5 *gene in traits related to hypertension and other components of metabolic syndrome. Our results suggest that *CAPN5 *alleles could modulate diastolic blood pressure and cholesterol levels. In addition, a *CAPN5 *haplotype over-represented in obese individuals is also associated with the cluster of cardiovascular risk factors defined as metabolic syndrome.

## Methods

### Patients

This population based study included 606 non-related Caucasian men (n = 278, 46%) and women (n = 328, 54%) recruited by a simple random sampling approach from a cross-sectional population-based epidemiological survey in the province of Segovia in Central Spain (Castille) aimed at investigating the prevalence of anthropometric and physiological parameters related to obesity and other components of the metabolic syndrome [20]. Clinical characteristics of study subjects are summarized in table 1. Individuals with previous diagnosis of type 1 diabetes were excluded from the study.

**Table 1:** Clinical characteristics of study subjects

	**Men (n = 278)**		**Women (n = 328)**	
	
	*Mean*	*SD*	*Mean*	*SD*
**Age (years)**	54.56	12.24	55.53	11.94
**BMI (kg/m^2^)**	27.47	3.42	27.41	4.58
**Waist circumference (cm)**	95.69	9.05	85.52	11.01
**SBP (mmHg)**	125.84	16.22	124.70	19.32
**DBP(mmHg)**	78.55	8.44	77.59	9.02
**Fasting glucose (mg/dl)**	92.79	29.78	87.74	24.05
**2 h-glucose (mg/dl)**	110.36	42.60	114.38	40.67
**Fasting insulin (mU/l)**	13.43	7.21	13.22	10.08
**HOMA**	3.08	1.97	2.99	3.08
**TGs (mg/dl)**	115.29	74.67	83.97	42.13
**Cholesterol (mg/dl)**	214.46	41.02	210.96	39.12
**HDL-c(mg/dl)**	55.64	16.55	64.85	18.64
**LDL-c (mg/dl)**	135.76	35.95	129.31	33.63

All study subjects gave their written consent to participate in the study. The study protocol was approved by the Ethics Committee of the Hospital Clinico San Carlos of Madrid.

### Phenotype measurements

We have analysed the existence of association between *CAPN5 *and twelve clinical parameters: body mass index (BMI), waist circumference (WC), systolic and diastolic blood pressures (SBP, DBP), fasting glucose, 2-hours glucose, fasting insulin, insulin resistance (IR-HOMA), total cholesterol, HDL-cholesterol, LDL-cholesterol and triglycerides (TGs).

SBP and DBP were measured three times in the seated position after 10 minutes of rest to the nearest even digit by use of a random-zero sphygmomanometer.

After an overnight fast period, 20 ml of blood were obtained from an antecubital vein without compression. Plasma glucose was determined in duplicate by a glucose-oxidase method adapted to an Autoanalyzer (Hitachi 704, Boehringer Mannheim, Germany). Total cholesterol, triglycerides and high-density lipoprotein (HDL) cholesterol were determined by enzymatic methods using commercial kits (Boehringer Mannheim, Germany). Low-density lipoprotein (LDL) cholesterol was calculated by the Friedewald formula. Serum insulin was determined by RIA (Human Insulin Specific RIA kit, Linco Research Inc., St Louis MO, USA).

Oral glucose tolerance test (OGTT) using 75 g of glucose was performed according to the WHO recommendations. Two hours after glucose administration, blood samples were obtained for the determination of glucose levels and interpreted the results according to Genuth et al. [21]. The crude prevalences of T2DM, impaired fasting glucose (IFG) and impaired glucose tolerance (IGT) were respectively 8.7%, 13.6% and 15.0%.

Insulin resistance was estimated by the homeostasis model assessment (HOMA-IR) method according to the formula: Insulin (mU/l) × Glucose (mmol/l)/22.5 [22].

The metabolic syndrome status was established according to ATPIII definition as it was recently modified: presence of at least three components between abdominal obesity (WC ≥ 102 cm men, 88 cm women), hypertriglyceridemia (TGs ≥ 150 mg/dl), hypertension (≥ 85/130 mmHg), HDL-c (< 40 mg/dl men, < 50 mg/dl women) and fasting glucose ≥ 100 mg/dl [23]. We have also classified subjects according to the recently International Diabetes Federation (IDF) worldwide definition of the metabolic syndrome for Europid populations [24]. In this last definition, central adiposity (defined as WC ≥ 94 cm for men and ≥ 80 cm for women) is a prerequisite risk factor for the diagnosis of the syndrome; two of the following factors are also necessary: raised TG level (>150 mg/dl) or specific treatment for this abnormality; reduced HDL-c (< 40 mg/dl males, < 50 mg/dl females) or specific treatment; raised blood pressure (SBP ≥ 130 or DBP ≥ 85) or treatment of previously diagnosed hypertension, raised fasting plasma glucose (FPG ≥ 100 mg/dl) or previously diagnosed T2DM. The crude prevalence of metabolic syndrome under ATPIII criteria is 12.6% and 19.8% under IDF definition.

### DNA extraction

DNA extraction from 5 ml of frozen peripheral blood was performed in a MagNa Pure LC Instrument (Roche Diagnostics), using MagNa Pure LC DNA Isolation kits (Roche Diagnostics) according to the manufacturer's instructions. Aliquots of DNA of 5 ng/ul were obtained to carry out PCR reactions.

### Genotyping

**Figure 1:**
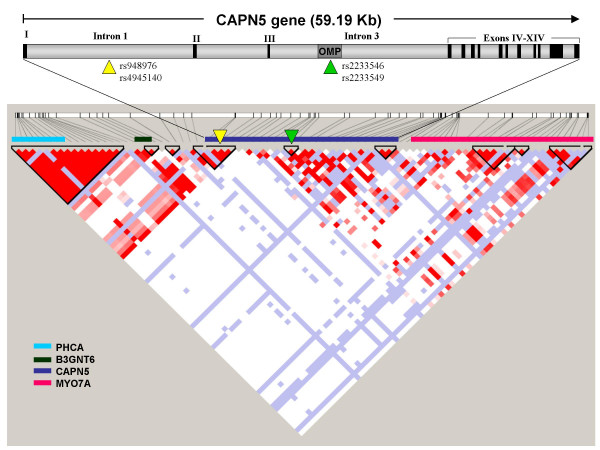
Scheme of *CAPN5 *gene organization and LD pattern of *CAPN5 *with neighboring genes (region spanning 140 kb aproximately). Analysed polymorphism are indicated by triangles.

We have analysed two polymorphisms in intron 1 (rs948976 A>G and rs4945140 G>A) and two polymorphisms located in the, intron 3 of *CAPN5 *gene (rs223546 C>T and rs223549 G>A) (Figure 1). These last two SNPs are located in the coding region of the OMP gene, an intronic gene integrated in the non-coding region of *CAPN5 *gene, so the aminoacids encoded by the OMP gene are not present in the CAPN5 protein. These SNPs were selected because they had been associated with PCOS, an insulin resistance related phenotype, in a previous work by our group [18]. The genotyping of the selected SNPs was performed according to described procedures [18].

### Statistical analysis

To analyze differences in genotype distribution, chi-squared association studies for metabolic syndrome, T2DM, IFG and IGT and test for deviation from Hardy-Weinberg equilibrium, we have used tests adapted from Sasieni [25] at the online resource available at the Institute for Human Genetics, Munich, Germany [26] or SPSS software (Ver. 11.0.0., LEAD Technologies, Inc).

For genotype quantitative analysis, we have performed an analysis of variance using the GLM procedure included in SPSS software (Ver. 11.0.0., LEAD Technologies, Inc). Normality of the dependent variables was assessed by Kolmogorov-Smirnov test and, when necessary, we applied mathematical transformations (natural logarithm or inverse of the square root) prior to the analysis. Each analysis was adjusted for age and sex. The adjusted percentage of the phenotypic variance explained by each genotype was estimated by subtracting the adjusted r2 value for a model that includes the genotype from the r2 for a model that excludes it. The Levene's test of equality of error variances was also performed for each analysis. In addition, all values from individuals under antihypertensive, antidiabetic or hypocholesterolemic medication were eliminated of the QTL analysis.

Linkage disequilibrium (LD) and haplotype analysis was performed using THESIAS software based on the SEM algorithm [27,28]. This method allows to estimate haplotype frequencies and haplotype effects by comparison to a reference (the intercept) taken here as the most frequent one. For quantitative analysis, haplotypes effects are expressed as increases/decreases of the phenotypic mean with respect to the intercept's one; for qualitative analysis, results are expressed as Odds ratio (OR). All the quantitative studies were corrected by age and sex. In a first approximation, all haplotypes identified were computed but, in order to minimize loss of power, haplotypes with a frequency lower than 3% were excluded from further analysis.

We have also performed a regression-based haplotype association test using Whap software [29,30] to confirm results obtained with the SEM approach. This software also allows executing a Monte-Carlo test to obtain the empirical p values. All values were standardized before the analysis according to software instructions.

## Results

### Allele frequencies and genotype distribution

Polymorphism status and allele frequencies were determined in all 606 individuals. The observed allelic frequencies in our population were 0.74 for A allele in rs948976 A>G, 0.57 for G allele in rs4945140 G>A, 0.94 for C allele in rs223546 C>T and 0.80 for G allele in rs223549G>A. The rs223546 is the less informative one, with only 0.06 frequency for the T allele and an observed heterozygosity of 0.11. All genotype frequencies fit the Hardy-Weinberg equilibrium (p ≥ 0.129). Genotype distribution and Pearson's goodness-of-fit chi-square test for deviation from Hardy-Weinberg equilibrium are shown in table 2.

**Table 2:** Genotype distribution and test for Hardy-Weinberg disequilibrium

SNP	F _AlLELE 1_	P _H-W(1 gl)_	GENOTYPE DISTRIBUTION		
rs948976 A>G	0.74	0.39	***AA***	***AG***	***GG***
			
			338 (55.8%)	224 (36.9%)	44 (7.3%)
rs4945140 G>A	0.57	0.40	***GG***	***GA***	***AA***
			
			200 (33.0%)	288 (47.5%)	118 (19.5%)
rs223546 C>T	0.94	1.0	***CC***	***CT***	***TT***
			
			536 (88.5%)	68 (11.2%)	2 (0.3%)
rs223549 G>A	0.80	0.13	***GG***	***GA***	***AA***
			
			391 (64.5%)	184 (30.4%)	31 (5.1%)

### Quantitative genotype analysis

In this analysis we have found significant differences in BMI values associated with the two markers of the first cluster (table 3). For the rs948976 A>G polymorphism, wild type homozygotes have the highest BMI values (means: AA, 27.4 ± 3.7; AG, 26.6 ± 4.1; GG, 26.8 ± 3.5 kg/m2; p = 0.035, r2 = 1.5%), whereas the presence of the polymorphic A allele at the rs4945140 G>A locus is associated with increased BMI values, especially in the homozygous state (means: GG, 26.5 ± 3.7; GA, 27.2 ± 3.8; AA, 27.8 ± 4.2 kg/m2; p = 0.041, r2 = 2.0%). The rs4945140 G>A marker is also associated with DBP values, being again the polymorphic A allele which confers increased risk (means: GG, 75.3 ± 8.7; GA, 77.7 ± 7.9; AA, 77.9 ± 7.8 mmHg; p = 0.015, r2 = 2.0%). Finally, HDL-c levels are associated with rs223549G>A genotypes; raised HDL-c values are related to the A allele (means: GG, 58.7 ± 17.2; GA, 64.0 ± 21.4; AA, 65.9 ± 18.8 mg/dl; p = 0.025, r2 = 1.7%). We have not find any other significant associations at genotype levels.

**Table 3:** Genotype analysis at the calpain 5 *locus*

PHENOTYPE	SNP	11	12	22	p	r^2^
BMI (kg/m2)	rs948976	27.4 ± 3.7	26.6 ± 4.1	26.8 ± 3.5	0.035	1.5%
	rs4945140	26.5 ± 3.7	27.2 ± 3.8	27.8 ± 4.2	0.041	2.0%
DBP (mmHg)	rs4945140	75.3 ± 8.7	77.7 ± 7.9	77.9 ± 7.8	0.015	2.0%
HDL-c (mg/dl)	rs223549	58.7 ± 17.2	64.0 ± 21.4	65.9 ± 18.8	0.025	1.7%

For each genotype, means and standard deviations are shown. rs948976, allele 1:allele A; rs4945140 allele 1, allele G; rs223549 allele 1, allele G.

### Haplotype analysis

The four polymorphisms analysed are in two LD blocks: rs948976 A>G and rs4945140 G>A in intron 1 (D' = -1, r^2 ^= 0.26) and rs223546 C>T and rs223549G>A in intron 3 (D' = -1, r^2 ^= 0.02), separated from each other by a recombination hot-spot (D' between blocks = 0, r2 = 7.8 × 10^-5^). With this LD pattern, nine different haplotypes are possible with frequencies ranging from 1.5% to 28.5% (table 4). The leading results of haplotype analysis are shown in table 5; the complete study is available online [see Additional files 1, 2, 3, 4, 5, 6, 7, 8, 9, 10, 11, 12, 13, 14, 15, 16, 17, 18, 19]. The highest global haplotypic effect was found for DBP (χ^2^_5d.f _= 14.96 with, p = 0.010) followed by total cholesterol (χ^2^_5d.f _= 11.43, p = 0.043). There is also a trend toward being associated for BMI, but it did not reach the statistical significance level (χ^2^_5d.f _= 10.8, p = 0.056). No significant global associations have been found for any of the other traits analysed (p ≥ 0.081).

**Table 4:** Haplotypes formed at *CAPN5 locus *with Nt g.86, Nt g.344, Nt c.1320 and Nt c.1469 polymorphisms

BASE SEQUENCE (rs948976A>G/rs4945140G>A/rs223546C>T/rs223546G>A)	POPULATION FREQUENCY
AA-CG	28.55%
AG-CG	25.2%
GG-CG	19.89%
AA-CA	12.04%
GG-CA	4.2%
AG-CA	4.05%
AA-TG	2.52%
AG-TG	1.89%
GG-TG	1.52%

**Table 5:** Effects of the main haplotypes of *CAPN5 *by comparison to the most frequent haplotype adjusted for age and sex

TRAIT	HAPLOTYPE	MEAN EFFECT [CI] (%)	P	χ^2^_5d.f_	P_GLOBAL_
BMI (kg/m2)	AA-CA	0.66 [-0.10 – 1.42] (+ 5.9%)	0.089	10.75	0.056
DBP (mmHg)	GG-CG	-2.22 [-3.82 – -0.62] (- 6.1%)	0.006	14.96	0.010
	AG-CA	-5.18 [-8.08 – -2.28] (- 14.2%)	0.0005		
Cholesterol (mg/dl)	GG-CG	-7.98 [-15.18 – -0.79] (- 8.0%)	0.029	11.43	0.043
	GG-CA	29.41 [11.66 – 47.17 ] (+ 29.6%)	0.001		

Haplotype effects are expressed as increases or decreases with CIs and as % over the reference haplotype mean.

According to the global haplotypic analysis, none of the *CAPN5 *haplotypes are consistently associated with BMI values and only the AA-CA haplotype shows a suggestive association (p = 0.086), being this result significant under de regression-based model (empirical p-value after 5.000 permutations = 0.039). We determined the frequency of the AA-CA haplotype in obese (BMI ≥ 30 kg/m^2^, 148 individuals) and non-obese subjects, being this haplotype over-represented in obese population (OR = 1.63 [1.05–2.54], p = 0.029). The haplotype analysis of BMI values in the two clusters independently (intron 1 from one side and intron 3 from another) showed that only the first cluster is informative for BMI (χ^2^_2d.f _6.07, p = 0.048) according to the genotype analysis. We have not found evidence of association of *CAPN5 *alleles with abdominal obesity estimated by waist circumference [see Additional file 2].

With respect to blood pressure, haplotypes AG-CA (p = 0.0005) and GG-CG (p = 0.006) are related to a significant decrease of DBP values (-14.2% and -6.1%, respectively). The, independent analysis of the two clusters showed that the second one is not significant whereas the global measure of association of cluster 1 is of a similar magnitude that in the full-length analysis (χ^2^_5d.f _= 7.84, p = 0.019)

Haplotype GG-CA is associated with higher total (p = 0.001, +29.6%) and LDL-cholesterol (p = 0.008, +36.1%). In contrast, haplotype GG-CG is related to a reduction in both values (p = 0.029, -8.0% and p = 0.044, -10.3%). None of the regions are independently associated with the trait (global p ≥ 0.090), suggesting that both are necessary to modulate the phenotype and explaining the absence of association in the genotype analysis. No associations have been found for HDL-c or TG levels in the haplotypic analysis [see Additional files 10 and 12].

We have not found evidence of *CAPN5 *being related to glucose or insulin levels [see Additional files 5, 6, 7]. Although the AG-CA haplotype was significantly associated with fasting glucose and 2 h-glucose levels (p ≤ 0.034), the global haplotypic effect was not significant (p ≥ 0.107). In this way, T2DM, IFG or IGT do not seem to be related to *CAPN5 *alleles, although the number of affected individuals is small (49, 77 and 74 individuals respectively) [see Additional files 15, 16, 17].

Considering metabolic syndrome under de ATPIII definition, we have found association with the obesity related AA-CA haplotype (p = 0.029, OR = 1.80 [1.06–3.06], n = 90), although the global haplotypic effect is not significant (p = 0.166). For the metabolic syndrome diagnosed following the IDF criteria, the global haplotypic effect was significant (χ^2^_5d.f _= 11.74, p = 0.039), but only a suggestive protective effect for the GG-CG haplotype could be determined (OR = 0.63 [0.37–1.08], p = 0.092).

## Discussion

In this population-based survey, we have isolated *CAPN5 *as a candidate gene for cardiovascular risk factors such as BMI, diastolic blood pressure and cholesterol. In addition, the haplotype related to obesity (AA-CA) is more frequent in individuals with metabolic syndrome (ATPIII criteria), supporting the role of obesity as an important underlying risk factor for cardiovascular diseases [31,32]. Two groups of haplotypes can be drawn regarding cardiovascular disease risk factors: protective haplotypes (GG-CG and AG-CA) and risk haplotypes (AA-CA and GG-CA).

In a previous study, we analysed these four *CAPN5 *gene variants in PCOS women, an anovulatory disorder of women in which insulin resistance plays a central role [33]. In that work, the GG-CA haplotype, associated with cholesterol levels in this work, was over-represented in PCOS patients. In addition, we associated several *CAPN5 *haplotypes with phenotypic differences observed between PCOS patients, such as the presence of obesity (AA-CA) and familial antecedents of obesity (AA-CA and GG-CG), hypertension (AA-CA and GG-CG) and hypercholesterolemia (GG-CA). [18]. Presence of familial antecedents of hypertension in PCOS women is a frequent finding; the higher frequency of this haplotype among women with familial antecedents of hypertension suggested a hypertensive phenotype associated to this allele. However, in this work, a measure of the effect of this haplotype in a population-based analysis has revealed a blood pressure lowering effect of the GG-CG haplotype. It is possible that smaller sample size in PCOS study, the effect of PCOS phenotype or PCOS related alleles have been acting as confounding factors. Anyway, the identification of *CAPN5 *gene haplotypes affecting cardiovascular risk factors also in general population reinforce *CAPN5 *as a candidate gene for metabolic syndrome related phenotypes. It is unlikely that the *OMP *gene itself be causative for the described associations in our population, given that haplotype association analysis at *OMP *region (intron 3) was not significant for any of the traits analysed, being necessary the *CAPN5 *intron 1 region to modulate the phenotype.

Little is known about CAPN5 function in humans. It is expressed in almost all human tissues, including testis, ovary, liver, heart, colon, kidney and brain [5][6]. Its homologue *CAPN10 *has been related to insulin secretion and action in both functional and genetic analyses [34]. Some authors have also pointed out a role for *CAPN10 *in adipocyte biology. Paul et al. [35] described how CAPN10 facilitates GLUT4 vesicle translocation during insulin-stimulated glucose uptake in adipocytes. Another report by Hoffstedt et al. [36] describes association of *CAPN10 *gene with the lipolytic sensitivity of *β*3-adrenoreceptors in subcutaneous fat cells in overweight subjects. Calpains have been also associated with adipocyte differentiation [37]. Regarding hypertension, calpains have been involved in mitochondrial mechanisms of oxidative stress that leads to vascular dysfunction which causes systemic hypertension and compensatory cardiac stiffness and diastolic heart failure [38].

The CAPN5 ortologhe in *C.elegans*, TRA-3, mediates the proteolitic cleavage of TRA-2A, a transmembrane protein that binds and inhibits the activity of the male-promoting FEM proteins (FEM-1, FEM-2 and FEM-3) [7]. The proteolitic action of TRA-3 over TRA-2 protein; produce a soluble fragment that is delivered to the nucleus where it enhances the transcriptional activity of the zinc finger transcription factor TRA-1 which promotes female development. Because mechanisms of sexual differentiation are not conserved between nematodes and human, it is possible that this pathway has more general functions than in *C.elegans*. In fact, the finding of abnormal glucose homeostasis in the *knockout *mice for Fem1b gene, the identification of a germline missense mutation in *FEM1A *gene in a PCOS patient and the observation of FEM1A being downregulated in human Rhabdomyosarcoma cell lines, support this hypothesis [8,9,39]. In humans, CAPN5 is localized to the cytoplasma as well as to the nucleus [40]. It is possible that, in a similar way of its ortologue TRA-3, CAPN5 could act over nuclear proteins that affect DNA transcription such as transcription factors or related proteins. Calpains are known to cleave several transcription factors: c-Fos, c-Jun, Oct-1, p53 or C/EBP transcription factor family among others, are calpain substrates [41-43]. Calpains can also affect DNA transcription in an indirect way. For example, activation of NF-kappa B requires the proteolitic degradation of its inhibitor I kappa B by calpains [44].

It is also possible that the identified associations are not related to *CAPN5 *gene itself, but other upstream gene in linkage disequilibrium with the polymorphic variants analysed. However, HapMap shows that LD block that includes the 5' region of *CAPN5 *is interrupted 29 kb upstream *CAPN5 *5'UTR. This region only includes the gene that codifies B3Gn-T6, a member of the N-acetylglucosaminyl transferase protein family related to muscular dystrophies, neoplastic disorders and altered glucose homeostasis, although B3Gn-T6 has never been analysed in humans [45]. The adjacent 5' LD block comprises 193 kb and includes the PHCA gene that codifies an alkaline phytoceramidase, an integral membrane protein related to protein biosynthesis and lipid membrane ceramide metabolism [46]. Beyond this region, is not probably to detect association with our marker selection. The absence of association in the independent cluster analysis of BMI, cholesterol levels and diastolic blood pressure with the markers of the second LD block that contains the OMP gene, makes improbable that the associations described here can be due to this intragenic gene. To elucidate this question, further analysis is being performed.

## Conclusion

Results obtained from our analysis in PCOS women and this population-based study suggest that *CAPN5 *is a gene related to metabolic syndrome and related phenotypes such as obesity, hypertension and hypercholesterolemia, at least in the Spanish population. These results are in accordance with the evidence of linkage of T2DM within the chromosomal region that contains *CAPN5 *gene reported by Silander *et al*. [19]. However, a confirmation of our findings in independent populations is necessary to clearly establish the role of *CAPN5 *in metabolic syndrome.

## Abbreviations

CVD: cardiovascular disease

T2DM: type 2 diabetes mellitus

MS: metabolic syndrome

CAPN10: calpain 10

CAPN5: calpain 5

PCOS: polycystic ovary syndrome

BMI: body mass index

WC: waist circumference

SBP: systolic blood pressure

DBP: diastolic blood pressure

IR: insulin resistance

HOMA: homeostasis model assessment

HDL: high density lipoprotein

LDL: low density lipoprotein

OGTT: oral glucose tolerance test

IFG: impaired fasting glucose

IGT: impaired glucose tolerance

TGs: triglycerides

LD: linkage disequilibrium

OR: odds ratio

## Competing interests

The authors ME Saez, R Ramirez-Lorca and A Ruiz have declared that conflicts of interest exist. Some of the work described here is subject to patent filings for diagnostics purposes (WO2005116247).

## Authors' contributions

MES: has genotyped samples, analysed and interpreted data and draft the manuscript.

RRL, FJM and AR: have contributed to conception and design of the study and revised the manuscript.

MTML, JLGL, CZ, MJMC and MSR: have contributed to the acquisition and interpretation of data and revised the manuscript.

All authors read and approved the final manuscript.

## Pre-publication history

The pre-publication history for this paper can be accessed here:



## Supplementary Material

Additional File 1 BMI. Haplotype association analysis of *CAPN5 *gene with BMI values using Thesias software. Click here for file

Additional File 2 Waist circumference. Haplotype association analysis of *CAPN5 *gene with waist circumference values using Thesias software. Click here for file

Additional File 3 SBP. Haplotype association analysis of *CAPN5 *gene with systolic blood pressure (SBP) values using Thesias software. Click here for file

Additional File 4 DBP. Haplotype association analysis of *CAPN5 *gene with diastolic blood pressure (DBP) values using Thesias software. Click here for file

Additional File 5 Fasting glucose. Haplotype association analysis of *CAPN5 *gene with fasting glucose values using Thesias software. Click here for file

Additional File 6 2 h-glucose. Haplotype association analysis of *CAPN5 *gene with glucose values after 2 hours from an oral glucose overload using Thesias software. Click here for file

Additional File 7 Fasting insulin. Haplotype association analysis of *CAPN5 *gene with fasting insulin values using Thesias software. Click here for file

Additional File 8 HOMA. Haplotype association analysis of *CAPN5 *gene with insulin resistance estimated using the Homeostasis Model Assessment method (HOMA) using Thesias software. Click here for file

Additional File 9 Cholesterol. Haplotype association analysis of *CAPN5 *gene with total cholesterol values using Thesias software. Click here for file

Additional File 10 HDL-c. Haplotype association analysis of *CAPN5 *gene with high-density lipoprotein cholesterol values using Thesias software. Click here for file

Additional File 11 LDL-c. Haplotype association analysis of *CAPN5 *gene with low-density lipoprotein cholesterol values using Thesias software. Click here for file

Additional File 12 TGs. Haplotype association analysis of *CAPN5 *gene with triglyceride values using Thesias software. Click here for file

Additional File 13 Obesity. Haplotype association analysis of *CAPN5 *gene with obesity defined as BMI ≥ 30 using Thesias software. Click here for file

Additional File 14 Obesity with hypertension. Haplotype association analysis of *CAPN5 *gene with obesity associated with hypertension (BMI ≥ 30 and SBP/DBP ≥ 130/85) using Thesias software. Click here for file

Additional File 15 T2DM. Haplotype association analysis of *CAPN5 *gene with Type 2 Diabetes Mellitus (T2DM) using Thesias software. Click here for file

Additional File 16 IFG. Haplotype association analysis of *CAPN5 *gene with Impaired Fasting Glucose (IFG) using Thesias software. Click here for file

Additional File 17 IGT. Haplotype association analysis of *CAPN5 *gene with Impaired Glucose Tolerance (IGT) using Thesias software. Click here for file

Additional File 18 SM(ATPIII). Haplotype association analysis of *CAPN5 *gene with Metabolic Syndrome using the ATPIII definition using Thesias software. Click here for file

Additional File 19 SM(IDF). Haplotype association analysis of *CAPN5 *gene with Metabolic Syndrome using the International Diabetes Federation (IDF) definition using Thesias software. Click here for file
