# Supplementary material for: Calpain-5 gene variants are associated with diastolic blood pressure and cholesterol levels
Source: BMC Med Genet. 2007 Jan 16;8:1. doi: 10.1186/1471-2350-8-1 (PMC1783645; doi:10.1186/1471-2350-8-1)
Supplement: Additional File 1 — BMI. Haplotype association analysis of CAPN5 gene with BMI values using Thesias software. [file 1471-2350-8-1-S1.doc]

| Haplotype Effects* |  |
| --- | --- |
| AACG | - (Intercept) |
| AGCG | Diff = 0.05652 [-0.55025 - 0.66329] p=0.855134 |
| GGCG | Diff = -0.42461 [-1.16104 - 0.31181] p=0.258430 |
| AACA | Diff = 0.66028 [-0.10271 - 1.42326] p=0.089855 |
| GGCA | Diff = -0.47196 [-1.64587 - 0.70196] p=0.430701 |
| AGCA | Diff = -1.39967 [-3.42617 - 0.62683] p=0.175819 |
|  | |
| Covariable Adjustment |  |
| Covariate 1 Age | Diff = 0.09015 [0.06365 - 0.11666] p=0.000000 |
| Covariate 2 Sex | Diff = -0.05629 [-0.72531 - 0.61274] p=0.869022 |
|  | |
| Polymorphism 1 A/G |  |
| Haplotypic Background -GCG | Diff = -0.48113 [-1.25723 - 0.29497] p=0.224338 |
| Haplotypic Background -GCA | Diff = 0.92772 [-1.60821 - 3.46364] p=0.473359 |
| Haplotypic Background -GTG | - |
| Haplotypic Background -ACG | - |
|  | |
| Polymorphism 2 G/A |  |
| Haplotypic Background A-CG | Diff = -0.05652 [-0.66329 - 0.55025] p=0.855134 |
| Haplotypic Background A-CA | Diff = 2.05995 [-0.05061 - 4.17051] p=0.055747 |
| Haplotypic Background A-TG | - |
| Haplotypic Background G-CG | - |
|  | |
| Polymorphism 3 C/T |  |
| Haplotypic Background AG-G | - |
| Haplotypic Background AA-G | - |
| Haplotypic Background GG-G | - |
|  | |
| Polymorphism 4 G/A |  |
| Haplotypic Background AGC- | Diff = -1.45619 [-3.59750 - 0.68512] p=0.182567 |
| Haplotypic Background AAC- | Diff = 0.66028 [-0.10271 - 1.42326] p=0.089855 |
| Haplotypic Background GGC- | Diff = -0.04734 [-1.48199 - 1.38730] p=0.948428 |
|  | |
| Expected Phenotypic Mean [95% CI] According to Estimated Haplotypes | |
| AACG | 11.12321 [10.18312 - 12.06330] |
| AGCG | 11.17973 [10.21534 - 12.14412] |
| GGCG | 10.69860 [9.61131 - 11.78589] |
| AACA | 11.78349 [10.64718 - 12.91980] |
| GGCA | 10.65125 [9.28949 - 12.01302] |
| AGCA | 9.72354 [7.49556 - 11.95152] |
| Global haplotypic effect: χ^2^ _5d.f_ =10.75; p=0.056 | |

* by comparison to the reference with its 95% CI (kg/m^2^).
