# Supplementary material for: Calpain-5 gene variants are associated with diastolic blood pressure and cholesterol levels
Source: BMC Med Genet. 2007 Jan 16;8:1. doi: 10.1186/1471-2350-8-1 (PMC1783645; doi:10.1186/1471-2350-8-1)
Supplement: Additional File 2 — Waist circumference. Haplotype association analysis of CAPN5 gene with waist circumference values using Thesias software. [file 1471-2350-8-1-S2.doc]

| Haplotype Effects* |  |
| --- | --- |
| AACG | - (Intercept) |
| AGCG | Diff = 0.45495 [-1.10445 - 2.01434] p=0.567443 |
| GGCG | Diff = -1.08810 [-2.84889 - 0.67269] p=0.225818 |
| AACA | Diff = 0.76710 [-1.23130 - 2.76549] p=0.451835 |
| GGCA | Diff = -1.46001 [-4.65465 - 1.73464] p=0.370384 |
| AGCA | Diff = -2.59220 [-6.94059 - 1.75618] p=0.242639 |
|  | |
| Covariable Adjustment |  |
| Covariate Age | Diff = 0.28803 [0.22549 - 0.35057] p=0.000000 |
| Covariate Sex | Diff = -10.37574 [-11.95725 - -8.79424] p=0.000000 |
|  | |
| Polymorphism 1 A/G |  |
| Haplotypic Background -GCG | Diff = -1.54305 [-3.38629 - 0.30020] p=0.100842 |
| Haplotypic Background -GCA | Diff = 1.13220 [-4.88148 - 7.14587] p=0.712120 |
| Haplotypic Background -GTG | - |
| Haplotypic Background -ACG | - |
|  | |
| Polymorphism 2 G/A |  |
| Haplotypic Background A-CG | Diff = -0.45495 [-2.01434 - 1.10445] p=0.567443 |
| Haplotypic Background A-CA | Diff = 3.35930 [-1.31520 - 8.03380] p=0.158971 |
| Haplotypic Background A-TG | - |
| Haplotypic Background G-CG | - |
|  | |
| Polymorphism 3 C/T |  |
| Haplotypic Background AG-G | - |
| Haplotypic Background AA-G | - |
| Haplotypic Background GG-G | - |
|  | |
| Polymorphism 4 G/A |  |
| Haplotypic Background AGC- | Diff = -3.04715 [-7.73420 - 1.63990] p=0.202579 |
| Haplotypic Background AAC- | Diff = 0.76710 [-1.23130 - 2.76549] p=0.451835 |
| Haplotypic Background GGC- | Diff = -0.37191 [-4.11193 - 3.36811] p=0.845469 |
|  | |
| Expected Phenotypic Mean [95% CI] According to Estimated Haplotypes | |
| AACG | 45.40121 [43.20702 - 47.59541] |
| AGCG | 45.85616 [43.63000 - 48.08232] |
| GGCG | 44.31312 [41.79522 - 46.83101] |
| AACA | 46.16831 [43.46311 - 48.87351] |
| GGCA | 43.94121 [40.34118 - 47.54123] |
| AGCA | 42.80901 [37.79377 - 47.82425] |
| Global haplotypic effect: χ^2^ _5d.f_ =7.28; p = 0.2006 | |

* by comparison to the reference with its 95% CI (cm).
