# Supplementary material for: Calpain-5 gene variants are associated with diastolic blood pressure and cholesterol levels
Source: BMC Med Genet. 2007 Jan 16;8:1. doi: 10.1186/1471-2350-8-1 (PMC1783645; doi:10.1186/1471-2350-8-1)
Supplement: Additional File 3 — SBP. Haplotype association analysis of CAPN5 gene with systolic blood pressure (SBP) values using Thesias software. [file 1471-2350-8-1-S3.doc]

| Haplotype Effects* |  |
| --- | --- |
| AACG | - (Intercept) |
| AGCG | Diff = 0.52703 [-2.67074 - 3.72479] p=0.746672 |
| GGCG | Diff = 0.05301 [-3.17341 - 3.27943] p=0.974309 |
| AACA | Diff = 0.42766 [-3.51194 - 4.36726] p=0.831510 |
| GGCA | Diff = -0.73071 [-8.24184 - 6.78043] p=0.848781 |
| AGCA | Diff = -0.23760 [-7.22823 - 6.75303] p=0.946887 |
|  | |
| Covariable Adjustment |  |
| Covariate 1 Age | Diff = 0.59874 [0.46271 - 0.73477] p=0.000000 |
| Covariate 2 Sex | Diff = -3.37763 [-6.17114 - -0.58412] p=0.017796 |
|  | |
| Polymorphism 1 A/G |  |
| Haplotypic Background -GCG | Diff = -0.47402 [-3.83829 - 2.89026] p=0.782426 |
| Haplotypic Background -GCA | Diff = -0.49311 [-12.18813 - 11.20192] p=0.934137 |
| Haplotypic Background -GTG | - |
|  | |
| Polymorphism 2 G/A |  |
| Haplotypic Background A-CG | Diff = -0.52703 [-3.72479 - 2.67074] p=0.746672 |
| Haplotypic Background A-CA | Diff = 0.66526 [-7.79811 - 9.12863] p=0.877559 |
| Haplotypic Background A-TG | - |
|  | |
| Polymorphism 3 C/T |  |
| Haplotypic Background AG-G | - |
| Haplotypic Background AA-G | - |
| Haplotypic Background GG-G | - |
|  | |
| Polymorphism 4 G/A |  |
| Haplotypic Background AGC- | Diff = -0.76463 [-8.30427 - 6.77501] p=0.842441 |
| Haplotypic Background AAC- | Diff = 0.42766 [-3.51194 - 4.36726] p=0.831510 |
| Haplotypic Background GGC- | Diff = -0.78372 [-8.94325 - 7.37582] p=0.850676 |
|  | |
| Expected Phenotypic Mean [95% CI] According to Estimated Haplotypes | |
| AACG | 36.77555 [29.82368 - 43.72742] |
| AGCG | 37.30258 [30.72034 - 43.88481] |
| GGCG | 36.82856 [29.62811 - 44.02901] |
| AACA | 37.20321 [29.41301 - 44.99340] |
| GGCA | 36.04484 [26.04278 - 46.04691] |
| AGCA | 36.53795 [26.73900 - 46.33690] |
| Global haplotypic effect: χ^2^ _5d.f_ =0.290, p=0.998 | |

* by comparison to the reference with its 95% CI (mmHg).
