# Supplementary material for: Calpain-5 gene variants are associated with diastolic blood pressure and cholesterol levels
Source: BMC Med Genet. 2007 Jan 16;8:1. doi: 10.1186/1471-2350-8-1 (PMC1783645; doi:10.1186/1471-2350-8-1)
Supplement: Additional File 4 — DBP. Haplotype association analysis of CAPN5 gene with diastolic blood pressure (DBP) values using Thesias software. [file 1471-2350-8-1-S4.doc]

| Haplotype Effects* |  |
| --- | --- |
| AACG | - (Intercept) |
| AGCG | Diff = -0.05501 [-1.40748 - 1.29745] p=0.936457 |
| GGCG | Diff = -2.22300 [-3.82580 - -0.62021] p=0.006559 |
| AACA | Diff = 0.04553 [-1.70979 - 1.80085] p=0.959453 |
| GGCA | Diff = 1.89464 [-1.50420 - 5.29348] p=0.274580 |
| AGCA | Diff = -5.18235 [-8.08439 - -2.28031] p=0.000465 |
|  | |
| Covariable Adjustment |  |
| Covariate 1 Age | Diff = 0.14954 [0.08841 - 0.21067] p=0.000002 |
| Covariate 2 Sex | Diff = -1.57944 [-3.03883 - -0.12004] p=0.033904 |
|  | |
| Polymorphism 1 A/G |  |
| Haplotypic Background -GCG | Diff = -2.16799 [-3.79018 - -0.54581] p=0.008807 |
| Haplotypic Background -GCA | Diff = 7.07699 [2.09563 - 12.05836] p=0.005360 |
| Haplotypic Background -GTG | - |
|  | |
| Polymorphism 2 G/A |  |
| Haplotypic Background A-CG | Diff = 0.05501 [-1.29745 - 1.40748] p=0.936457 |
| Haplotypic Background A-CA | Diff = 5.22788 [1.88501 - 8.57076] p=0.002175 |
| Haplotypic Background A-TG | - |
|  | |
| Polymorphism 3 C/T |  |
| Haplotypic Background AG-G | - |
| Haplotypic Background AA-G | - |
| Haplotypic Background GG-G | - |
|  | |
| Polymorphism 4 G/A |  |
| Haplotypic Background AGC- | Diff = -5.12734 [-8.26027 - -1.99441] p=0.001338 |
| Haplotypic Background AAC- | Diff = 0.04553 [-1.70979 - 1.80085] p=0.959453 |
| Haplotypic Background GGC- | Diff = 4.11765 [0.39686 - 7.83843] p=0.030079 |
|  | |
| Expected Phenotypic Mean [95% CI] According to Estimated Haplotypes | |
| AACG | 36.25146 [33.93364 - 38.56927] |
| AGCG | 36.19645 [34.06581 - 38.32709] |
| GGCG | 34.02845 [31.64717 - 36.40973] |
| AACA | 36.29699 [33.69418 - 38.89979] |
| GGCA | 38.14610 [34.30589 - 41.98630] |
| AGCA | 31.06910 [27.46050 - 34.67771] |
| Global haplotypic effect: χ^2^ _5d.f_ =14.96; p=0.010 | |

* by comparison to the reference with its 95% CI (mmHg).
