# Supplementary material for: Calpain-5 gene variants are associated with diastolic blood pressure and cholesterol levels
Source: BMC Med Genet. 2007 Jan 16;8:1. doi: 10.1186/1471-2350-8-1 (PMC1783645; doi:10.1186/1471-2350-8-1)
Supplement: Additional File 5 — Fasting glucose. Haplotype association analysis of CAPN5 gene with fasting glucose values using Thesias software. [file 1471-2350-8-1-S5.doc]

| Haplotype Effects* |  |
| --- | --- |
| AACG | - (Intercept) |
| AGCG | Diff = -1.07253 [-6.96735 - 4.82229] p=0.721383 |
| GGCG | Diff = -1.76754 [-8.78032 - 5.24523] p=0.621299 |
| AACA | Diff = 0.15139 [-9.50709 - 9.80987] p=0.975491 |
| AGCA | Diff = 11.93201 [4.15633 - 19.70769] p=0.002633 |
| GGCA | Diff = 2.19184 [-6.85307 - 11.23674] p=0.634813 |
|  | |
| Covariable Adjustment |  |
| Covariate 1 Age | Diff = 0.17733 [-0.11009 - 0.46476] p=0.226558 |
| Covariate 2 Sex | Diff = -7.24857 [-13.61400 - -0.88314] p=0.025620 |
|  | |
| Polymorphism 1 A/G |  |
| Haplotypic Background -GCG | Diff = -0.69501 [-7.87361 - 6.48358] p=0.849496 |
| Haplotypic Background -GCA | Diff = -9.74018 [-19.14521 - -0.33514] p=0.042372 |
| Haplotypic Background -GTG | - |
|  | |
| Polymorphism 2 G/A |  |
| Haplotypic Background A-CG | Diff = 1.07253 [-4.82229 - 6.96735] p=0.721383 |
| Haplotypic Background A-CA | Diff = -11.78062 [-23.03856 - -0.52268] p=0.040267 |
| Haplotypic Background A-TG | - |
|  | |
| Polymorphism 3 C/T |  |
| Haplotypic Background AG-G | - |
| Haplotypic Background AA-G | - |
| Haplotypic Background GG-G | - |
|  | |
| Polymorphism 4 G/A |  |
| Haplotypic Background AGC- | Diff = 13.00454 [4.26187 - 21.74721] p=0.003552 |
| Haplotypic Background AAC- | Diff = 0.15139 [-9.50709 - 9.80987] p=0.975491 |
| Haplotypic Background GGC- | Diff = 3.95938 [-5.83065 - 13.74941] p=0.427963 |
|  | |
| Expected Phenotypic Mean [95% CI] According to Estimated Haplotypes | |
|  |  |
| AACG | 44.41452 [34.55448 - 54.27456] |
| AGCG | 43.34199 [34.13793 - 52.54606] |
| GGCG | 42.64698 [33.29107 - 52.00289] |
| AACA | 44.56591 [32.01668 - 57.11515] |
| AGCA | 56.34653 [48.20795 - 64.48512] |
| GGCA | 46.60636 [36.09114 - 57.12157] |
| Global haplotypic effect: χ^2^ _5d.f_ =0.784, p=0.165 | |

* by comparison to the reference with its 95% CI (mg/dl).
