# Supplementary material for: Calpain-5 gene variants are associated with diastolic blood pressure and cholesterol levels
Source: BMC Med Genet. 2007 Jan 16;8:1. doi: 10.1186/1471-2350-8-1 (PMC1783645; doi:10.1186/1471-2350-8-1)
Supplement: Additional File 6 — 2 h-glucose. Haplotype association analysis of CAPN5 gene with glucose values after 2 hours from an oral glucose overload using Thesias software. [file 1471-2350-8-1-S6.doc]

| Haplotype Effects* |  |
| --- | --- |
| AACG | - (Intercept) |
| AGCG | Diff = -6.72963 [-13.95137 - 0.49210] p=0.067784 |
| GGCG | Diff = -4.07719 [-11.78512 - 3.63074] p=0.299847 |
| AACA | Diff = -10.88489 [-21.58082 - -0.18897] p=0.046083 |
| AGCA | Diff = 12.79604 [0.94572 - 24.64637] p=0.034309 |
| GGCA | Diff = -12.05562 [-33.78726 - 9.67602] p=0.276900 |
|  | |
| Covariable Adjustment |  |
| Covariate 1 Age | Diff = 0.87723 [0.56716 - 1.18730] p=0.000000 |
| Covariate 2 Sex | Diff = 0.37793 [-6.75405 - 7.50991] p=0.917279 |
|  | |
| Polymorphism 1 A/G |  |
| Haplotypic Background -GCG | Diff = 2.65245 [-5.74974 - 11.05464] p=0.536086 |
| Haplotypic Background -GCA | Diff = -24.85166 [-49.63607 - -0.06725] p=0.049377 |
| Haplotypic Background -GTG | - |
|  | |
| Polymorphism 2 G/A |  |
| Haplotypic Background A-CG | Diff = 6.72963 [-0.49210 - 13.95137] p=0.067784 |
| Haplotypic Background A-CA | Diff = -23.68093 [-38.60066 - -8.76121] p=0.001865 |
| Haplotypic Background A-TG | - |
|  | |
| Polymorphism 3 C/T |  |
| Haplotypic Background AG-G | - |
| Haplotypic Background AA-G | - |
| Haplotypic Background GG-G | - |
|  | |
| Polymorphism 4 G/A |  |
| Haplotypic Background AGC- | Diff = 19.52568 [6.52440 - 32.52696] p=0.003244 |
| Haplotypic Background AAC- | Diff = -10.88489 [-21.58082 - -0.18897] p=0.046083 |
| Haplotypic Background GGC- | Diff = -7.97843 [-31.95649 - 15.99963] p=0.514293 |
|  | |
| Expected Phenotypic Mean [95% CI] According to Estimated Haplotypes | |
| AACG | 34.63051 [23.68173 - 45.57929] |
| AGCG | 27.90087 [17.63127 - 38.17048] |
| GGCG | 30.55332 [19.51331 - 41.59333] |
| AACA | 23.74562 [9.91536 - 37.57587] |
| AGCA | 47.42655 [31.82669 - 63.02641] |
| GGCA | 22.57489 [-1.06316 - 46.21294] |
| Global haplotypic effect: χ^2^ _5d.f_ =9.04, p=0.107 | |

* by comparison to the reference with its 95% CI (mg/dl).
