# Supplementary material for: Calpain-5 gene variants are associated with diastolic blood pressure and cholesterol levels
Source: BMC Med Genet. 2007 Jan 16;8:1. doi: 10.1186/1471-2350-8-1 (PMC1783645; doi:10.1186/1471-2350-8-1)
Supplement: Additional File 7 — Fasting insulin. Haplotype association analysis of CAPN5 gene with fasting insulin values using Thesias software. [file 1471-2350-8-1-S7.doc]

| Haplotype Effects* |  |
| --- | --- |
| AACG | - (Intercept) |
| AGCG | Diff = 0.26400 [-1.40181 - 1.92981] p=0.756087 |
| GGCG | Diff = -0.36034 [-1.98395 - 1.26327] p=0.663563 |
| AACA | Diff = 0.15130 [-2.72012 - 3.02271] p=0.917746 |
| GGCA | Diff = 1.16648 [-2.34268 - 4.67565] p=0.514709 |
| AGCA | Diff = 1.14635 [-2.79620 - 5.08889] p=0.568748 |
|  | |
| Covariable Adjustment |  |
| Covariate 1 Age | Diff = 0.06377 [-0.01374 - 0.14128] p=0.106839 |
| Covariate 2 Sex | Diff = -0.24326 [-1.88438 - 1.39786] p=0.771412 |
|  | |
| Polymorphism 1 A/G |  |
| Haplotypic Background -GCG | Diff = -0.62434 [-2.33942 - 1.09074] p=0.475537 |
| Haplotypic Background -GCA | Diff = 0.02013 [-5.60425 - 5.64451] p=0.994402 |
| Haplotypic Background -GTG | - |
|  | |
| Polymorphism 2 G/A |  |
| Haplotypic Background A-CG | Diff = -0.26400 [-1.92981 - 1.40181] p=0.756087 |
| Haplotypic Background A-CA | Diff = -0.99505 [-5.69589 - 3.70579] p=0.678227 |
| Haplotypic Background A-TG | - |
|  | |
| Polymorphism 3 C/T |  |
| Haplotypic Background AG-G | - |
| Haplotypic Background AA-G | - |
| Haplotypic Background GG-G | - |
|  | |
| Polymorphism 4 G/A |  |
| Haplotypic Background AGC- | Diff = 0.88235 [-3.42859 - 5.19329] p=0.688298 |
| Haplotypic Background AAC- | Diff = 0.15130 [-2.72012 - 3.02271] p=0.917746 |
| Haplotypic Background GGC- | Diff = 1.52682 [-2.26425 - 5.31790] p=0.429894 |
|  | |
| Expected Phenotypic Mean [95% CI] According to Estimated Haplotypes | |
| AACG | 4.53487 [2.06179 - 7.00796] |
| AGCG | 4.79887 [2.32687 - 7.27087] |
| GGCG | 4.17453 [1.78133 - 6.56773] |
| AACA | 4.68617 [1.40238 - 7.96996] |
| GGCA | 5.70135 [1.81280 - 9.58991] |
| AGCA | 5.68122 [1.31621 - 10.04623] |
| Global haplotypic effect: χ^2^ _5d.f_ =1.73, p=0.885 | |

* by comparison to the reference with its 95% CI (mU/l).
