# Supplementary material for: Calpain-5 gene variants are associated with diastolic blood pressure and cholesterol levels
Source: BMC Med Genet. 2007 Jan 16;8:1. doi: 10.1186/1471-2350-8-1 (PMC1783645; doi:10.1186/1471-2350-8-1)
Supplement: Additional File 8 — HOMA. Haplotype association analysis of CAPN5 gene with insulin resistance estimated using the Homeostasis Model Assessment method (HOMA) using Thesias software. [file 1471-2350-8-1-S8.doc]

| Haplotype Effects* |  |
| --- | --- |
| AACG | - (Intercept) |
| AGCG | Diff = 0.08720 [-0.41030 - 0.58471] p=0.731186 |
| GGCG | Diff = 0.05058 [-0.42281 - 0.52397] p=0.834112 |
| AACA | Diff = -0.12387 [-0.98184 - 0.73410] p=0.777189 |
| GGCA | Diff = -0.23276 [-2.06374 - 1.59822] p=0.803238 |
| AGCA | Diff = 0.60111 [-0.67347 - 1.87570] p=0.355296 |
|  | |
| Covariable Adjustment |  |
| Covariate 1 Age | Diff = 0.02716 [0.00207 - 0.05225] p=0.033831 |
| Covariate 2 Sex | Diff = -0.12614 [-0.60351 - 0.35122] p=0.604510 |
|  | |
| Polymorphism 1 A/G |  |
| Haplotypic Background -GCG | Diff = -0.03662 [-0.51643 - 0.44319] p=0.881089 |
| Haplotypic Background -GCA | Diff = -0.83387 [-3.35533 - 1.68759] p=0.516861 |
| Haplotypic Background -GTG | - |
|  | |
| Polymorphism 2 G/A |  |
| Haplotypic Background A-CG | Diff = -0.08720 [-0.58471 - 0.41030] p=0.731186 |
| Haplotypic Background A-CA | Diff = -0.72499 [-2.25896 - 0.80898] p=0.354270 |
| Haplotypic Background A-TG | - |
|  | |
| Polymorphism 3 C/T |  |
| Haplotypic Background AG-G | - |
| Haplotypic Background AA-G | - |
| Haplotypic Background GG-G | - |
|  | |
| Polymorphism 4 G/A |  |
| Haplotypic Background AGC- | Diff = 0.51391 [-0.83971 - 1.86753] p=0.456800 |
| Haplotypic Background AAC- | Diff = -0.12387 [-0.98184 - 0.73410] p=0.777189 |
| Haplotypic Background GGC- | Diff = -0.28334 [-2.20819 - 1.64151] p=0.772954 |
|  | |
| Expected Phenotypic Mean [95% CI] According to Estimated Haplotypes | |
| AACG | 0.47914 [-0.35352 - 1.31181] |
| AGCG | 0.56635 [-0.20702 - 1.33971] |
| GGCG | 0.52973 [-0.22997 - 1.28943] |
| AACA | 0.35527 [-0.65357 - 1.36411] |
| GGCA | 0.24639 [-1.69018 - 2.18295] |
| AGCA | 1.08026 [-0.31612 - 2.47664] |
| Global haplotypic effect: χ^2^ _5d.f_ =2.45, p=0.784 | |

* by comparison to the reference with its 95% CI.
