# Supplementary material for: Calpain-5 gene variants are associated with diastolic blood pressure and cholesterol levels
Source: BMC Med Genet. 2007 Jan 16;8:1. doi: 10.1186/1471-2350-8-1 (PMC1783645; doi:10.1186/1471-2350-8-1)
Supplement: Additional File 9 — Cholesterol. Haplotype association analysis of CAPN5 gene with total cholesterol values using Thesias software. [file 1471-2350-8-1-S9.doc]

| Haplotype Effects* |  |
| --- | --- |
| AACG | - (Intercept) |
| AGCG | Diff = -4.14791 [-11.70184 - 3.40602] p=0.281816 |
| GGCG | Diff = -7.98559 [-15.17947 - -0.79171] p=0.029577 |
| AACA | Diff = -2.12303 [-11.57583 - 7.32977] p=0.659791 |
| AGCA | Diff = -4.48706 [-18.68163 - 9.70752] p=0.535536 |
| GGCA | Diff = 29.41270 [11.65651 - 47.16889] p=0.001168 |
|  | |
| Covariable Adjustment |  |
| Covariate 1 Age | Diff = 0.37905 [0.06932 - 0.68878] p=0.016455 |
| Covariate 2 Sex | Diff = -2.08172 [-9.35840 - 5.19495] p=0.574988 |
|  | |
| Polymorphism 1 A/G |  |
| Haplotypic Background -GCG | Diff = -3.83768 [-11.31724 - 3.64188] p=0.314582 |
| Haplotypic Background -GCA | Diff = 33.89976 [8.57074 - 59.22878] p=0.008710 |
| Haplotypic Background -GTG | - |
|  | |
| Polymorphism 2 G/A |  |
| Haplotypic Background A-CG | Diff = 4.14791 [-3.40602 - 11.70184] p=0.281816 |
| Haplotypic Background A-CA | Diff = 2.36403 [-15.27220 - 20.00025] p=0.792762 |
| Haplotypic Background A-TG | - |
|  | |
| Polymorphism 3 C/T |  |
| Haplotypic Background AG-G | - |
| Haplotypic Background AA-G | - |
| Haplotypic Background GG-G | - |
|  | |
| Polymorphism 4 G/A |  |
| Haplotypic Background AGC- | Diff = -0.33915 [-15.52531 - 14.84702] p=0.965086 |
| Haplotypic Background AAC- | Diff = -2.12303 [-11.57583 - 7.32977] p=0.659791 |
| Haplotypic Background GGC- | Diff = 37.39829 [19.66809 - 55.12850] p=0.000036 |
|  | |
| Expected Phenotypic Mean [95% CI] According to Estimated Haplotypes | |
| AACG | 99.39649 [87.94771 - 110.84527] |
| AGCG | 95.24858 [84.96765 - 105.52951] |
| GGCG | 91.41090 [80.25107 - 102.57074] |
| AACA | 97.27346 [84.60945 - 109.93748] |
| AGCA | 94.90944 [77.44911 - 112.36976] |
| GGCA | 128.80920 [110.32138 - 147.29701] |
| Global haplotypic effect: χ^2^ _5d.f_ =11.43, p=0.043 | |

* by comparison to the reference with its 95% CI (mg/dl).
