# Supplementary material for: Calpain-5 gene variants are associated with diastolic blood pressure and cholesterol levels
Source: BMC Med Genet. 2007 Jan 16;8:1. doi: 10.1186/1471-2350-8-1 (PMC1783645; doi:10.1186/1471-2350-8-1)
Supplement: Additional File 10 — HDL-c. Haplotype association analysis of CAPN5 gene with high-density lipoprotein cholesterol values using Thesias software. [file 1471-2350-8-1-S10.doc]

| Haplotype Effects* |  |
| --- | --- |
| AACG | - (Intercept) |
| AGCG | Diff = -0.56443 [-4.24288 - 3.11402] p=0.763607 |
| GGCG | Diff = -1.41597 [-5.11079 - 2.27886] p=0.452574 |
| AACA | Diff = 2.62688 [-2.11914 - 7.37290] p=0.277991 |
| AGCA | Diff = 3.96680 [-2.65324 - 10.58685] p=0.240214 |
| GGCA | Diff = 8.18365 [1.06910 - 15.29820] p=0.024163 |
|  | |
| Covariable Adjustment |  |
| Covariate 1 Age | Diff = -0.05511 [-0.20799 - 0.09778] p=0.479899 |
| Covariate 2 Sex | Diff = 9.85178 [6.35906 - 13.34450] p=0.000000 |
|  | |
| Polymorphism 1 A/G |  |
| Haplotypic Background -GCG | Diff = -0.85154 [-4.84941 - 3.14634] p=0.676331 |
| Haplotypic Background -GCA | Diff = 4.21685 [-6.41230 - 14.84600] p=0.436816 |
| Haplotypic Background -GTG | - |
|  | |
| Polymorphism 2 G/A |  |
| Haplotypic Background A-CG | Diff = 0.56443 [-3.11402 - 4.24288] p=0.763607 |
| Haplotypic Background A-CA | Diff = -1.33992 [-9.71183 - 7.03200] p=0.753752 |
| Haplotypic Background A-TG | - |
|  | |
| Polymorphism 3 C/T |  |
| Haplotypic Background AG-G | - |
| Haplotypic Background AA-G | - |
| Haplotypic Background GG-G | - |
|  | |
| Polymorphism 4 G/A |  |
| Haplotypic Background AGC- | Diff = 4.53123 [-3.16838 - 12.23084] p=0.248720 |
| Haplotypic Background AAC- | Diff = 2.62688 [-2.11914 - 7.37290] p=0.277991 |
| Haplotypic Background GGC- | Diff = 9.59962 [1.64357 - 17.55566] p=0.018035 |
|  | |
| Expected Phenotypic Mean [95% CI] According to Estimated Haplotypes | |
| AACG | 23.96177 [18.49297 - 29.43057] |
| AGCG | 23.39734 [18.04527 - 28.74941] |
| GGCG | 22.54580 [16.80657 - 28.28504] |
| AACA | 26.58865 [20.80958 - 32.36773] |
| AGCA | 27.92857 [19.46281 - 36.39433] |
| GGCA | 32.14542 [23.96074 - 40.33010] |
| Global haplotypic effect: χ^2^ _5d.f_ =9.45, p=0.092 | |

* by comparison to the reference with its 95% CI (mg/dl).
