# Supplementary material for: Calpain-5 gene variants are associated with diastolic blood pressure and cholesterol levels
Source: BMC Med Genet. 2007 Jan 16;8:1. doi: 10.1186/1471-2350-8-1 (PMC1783645; doi:10.1186/1471-2350-8-1)
Supplement: Additional File 11 — LDL-c. Haplotype association analysis of CAPN5 gene with low-density lipoprotein cholesterol values using Thesias software. [file 1471-2350-8-1-S11.doc]

| Haplotype Effects* |  |
| --- | --- |
| AACG | - (Intercept) |
| AGCG | Diff = -3.62299 [-9.77388 - 2.52790] p=0.248304 |
| GGCG | Diff = -6.36957 [-12.57105 - -0.16809] p=0.044101 |
| AACA | Diff = -5.04748 [-13.71441 - 3.61945] p=0.253673 |
| AGCA | Diff = -9.62498 [-22.71380 - 3.46384] p=0.149499 |
| GGCA | Diff = 22.38374 [5.84765 - 38.91983] p=0.007975 |
|  |  |
| Covariable Adjustment |  |
| Covariate 1 Age | Diff = 0.37240 [0.10353 - 0.64126] p=0.006632 |
| Covariate 2 Sex | Diff = -4.99096 [-11.26777 - 1.28585] p=0.119120 |
|  |  |
| Polymorphism 1 A/G |  |
| Haplotypic Background -GCG | Diff = -2.74658 [-9.25988 - 3.76673] p=0.408517 |
| Haplotypic Background -GCA | Diff = 32.00871 [8.60442 - 55.41301] p=0.007349 |
| Haplotypic Background -GTG | - |
|  | |
| Polymorphism 2 G/A |  |
| Haplotypic Background A-CG | Diff = 3.62299 [-2.52790 - 9.77388] p=0.248304 |
| Haplotypic Background A-CA | Diff = 4.57750 [-12.21344 - 21.36844] p=0.593114 |
| Haplotypic Background A-TG | - |
|  | |
| Polymorphism 3 C/T |  |
| Haplotypic Background AG-G | - |
| Haplotypic Background AA-G | - |
| Haplotypic Background GG-G | - |
|  | |
| Polymorphism 4 G/A |  |
| Haplotypic Background AGC- | Diff = -6.00199 [-20.06290 - 8.05893] p=0.402796 |
| Haplotypic Background AAC- | Diff = -5.04748 [-13.71441 - 3.61945] p=0.253673 |
| Haplotypic Background GGC- | Diff = 28.75330 [11.81049 - 45.69612] p=0.000880 |
|  | |
| Expected Phenotypic Mean [95% CI] According to Estimated Haplotypes | |
| AACG | 61.98756 [52.19826 - 71.77685] |
| AGCG | 58.36456 [49.40333 - 67.32580] |
| GGCG | 55.61799 [45.65363 - 65.58234] |
| AACA | 56.94008 [45.95530 - 67.92485] |
| AGCA | 52.36258 [36.30221 - 68.42294] |
| GGCA | 84.37129 [67.10507 - 101.63752] |
| Global haplotypic effect: χ^2^ _5d.f_ =9.80, p=0.081 | |

* by comparison to the reference with its 95% CI (mg/dl).
