# Supplementary material for: Calpain-5 gene variants are associated with diastolic blood pressure and cholesterol levels
Source: BMC Med Genet. 2007 Jan 16;8:1. doi: 10.1186/1471-2350-8-1 (PMC1783645; doi:10.1186/1471-2350-8-1)
Supplement: Additional File 12 — TGs. Haplotype association analysis of CAPN5 gene with triglyceride values using Thesias software. [file 1471-2350-8-1-S12.doc]

| Haplotype Effects* |  |
| --- | --- |
| AACG | Intercept 1 |
| AGCG | Diff = -1.90012 [-11.14855 - 7.34832] p=0.687178 |
| GGCG | Diff = 0.64454 [-11.30967 - 12.59875] p=0.915838 |
| AACA | Diff = -0.62332 [-17.68489 - 16.43825] p=0.942915 |
| AGCA | Diff = 19.49303 [-0.39050 - 39.37656] p=0.054668 |
| GGCA | Diff = -17.78008 [-46.98553 - 11.42537] p=0.232777 |
|  | |
| Covariable Adjustment |  |
| Covariate 1 Age | Diff = 0.31576 [-0.18661 - 0.81813] p=0.217968 |
| Covariate 2 Sex | Diff = -35.17984 [-48.29536 - -22.06433] p=0.000000 |
|  | |
| Polymorphism 1 A/G |  |
| Haplotypic Background -GCG | Diff = 2.54466 [-9.57947 - 14.66878] p=0.680799 |
| Haplotypic Background -GCA | Diff = -37.27311 [-72.43340 - -2.11282] p=0.037730 |
| Haplotypic Background -GTG | - |
|  | |
| Polymorphism 2 G/A |  |
| Haplotypic Background A-CG | Diff = 1.90012 [-7.34832 - 11.14855] p=0.687178 |
| Haplotypic Background A-CA | Diff = -20.11636 [-47.36392 - 7.13121] p=0.147888 |
| Haplotypic Background A-TG | - |
|  | |
| Polymorphism 3 C/T |  |
| Haplotypic Background AG-G | - |
| Haplotypic Background AA-G | - |
| Haplotypic Background GG-G | - |
|  | |
| Polymorphism 4 G/A |  |
| Haplotypic Background AGC- | Diff = 21.39315 [0.45063 - 42.33566] p=0.045266 |
| Haplotypic Background AAC- | Diff = -0.62332 [-17.68489 - 16.43825] p=0.942915 |
| Haplotypic Background GGC- | Diff = -18.42462 [-49.37277 - 12.52353] p=0.243266 |
|  | |
| Expected Phenotypic Mean [95% CI] According to Estimated Haplotypes | |
| AACG | 66.76833 [50.73460 - 82.80205] |
| AGCG | 64.86821 [49.69896 - 80.03746] |
| GGCG | 67.41287 [49.13120 - 85.69454] |
| AACA | 66.14500 [44.65649 - 87.63351] |
| AGCA | 86.26136 [62.15829 - 110.36443] |
| GGCA | 48.98825 [15.79488 - 82.18161] |
| Global haplotypic effect: χ^2^ _5d.f_ =3.11, p=0.683 | |

* by comparison to the reference with its 95% CI (mg/dl).
