# Supplementary material for: Calpain-5 gene variants are associated with diastolic blood pressure and cholesterol levels
Source: BMC Med Genet. 2007 Jan 16;8:1. doi: 10.1186/1471-2350-8-1 (PMC1783645; doi:10.1186/1471-2350-8-1)
Supplement: Additional File 13 — Obesity. Haplotype association analysis of CAPN5 gene with obesity defined as BMI ≥ 30 using Thesias software. [file 1471-2350-8-1-S13.doc]

| Haplotype Effects* |  | |
| --- | --- | --- |
| AACG | - (Intercept) | |
| AGCG | OR = 1.19874 [0.83064 - 1.72997] p=0.332762 | |
| GGCG | OR = 0.98344 [0.63684 - 1.51868] p=0.939972 | |
| AACA | OR = 1.63374 [1.05040 - 2.54106] p=0.029394 | |
| GGCA | OR = 1.30449 [0.60535 - 2.81112] p=0.497402 | |
| AGCA | OR = 0.22055 [0.05184 - 0.93821] p=0.040722 | |
|  | | |
| Polymorphism 1 G/A |  | |
| Haplotypic Background -GCG | OR = 1.21893 [0.79427 - 1.87063] p=0.364963 | |
| Haplotypic Background -GCA | OR = 0.16907 [0.03156 - 0.90572] p=0.037928 | |
| Haplotypic Background -GTG | - | |
| Haplotypic Background -ACG | - | |
|  | | |
| Polymorphism 2 G/A |  | |
| Haplotypic Background G-CG | - | |
| Haplotypic Background A-CG | OR = 0.83421 [0.57805 - 1.20389] p=0.332762 | |
| Haplotypic Background A-CA | OR = 7.40771 [1.66130 - 33.03090] p=0.008652 | |
| Haplotypic Background A-TG | - | |
|  | | |
| Polymorphism 3 C/T |  | |
| Haplotypic Background GG-G | - | |
| Haplotypic Background AG-G | - | |
| Haplotypic Background AA-G | - | |
|  | | |
| Polymorphism 4 G/A |  | |
| Haplotypic Background GGC- | OR = 1.32645 [0.53689 - 3.27719] p=0.540407 | |
| Haplotypic Background AGC- | OR = 0.18398 [0.04034 - 0.83914] p=0.028779 | |
| Haplotypic Background AAC- | OR = 1.63374 [1.05040 - 2.54106] p=0.029394 | |
|  |  | |
| Haplotype frequencies | Controls (n=452) | Cases (n=148) |
| AACG | 0.289045 | 0.271277 |
| AGCG | 0.248914 | 0.266855 |
| GGCG | 0.201749 | 0.184473 |
| AACA | 0.106064 | 0.165760 |
| GGCA | 0.042500 | 0.046145 |
| AGCA | 0.048338 | 0.011068 |
| Global haplotypic effect: χ^2^ _5d.f_ =10.75, p=0.056 | | |

* Haplotypic OR by comparison to the reference with its 95% CI
