# Supplementary material for: Calpain-5 gene variants are associated with diastolic blood pressure and cholesterol levels
Source: BMC Med Genet. 2007 Jan 16;8:1. doi: 10.1186/1471-2350-8-1 (PMC1783645; doi:10.1186/1471-2350-8-1)
Supplement: Additional File 14 — Obesity with hypertension. Haplotype association analysis of CAPN5 gene with obesity associated with hypertension (BMI ≥ 30 and SBP/DBP ≥ 130/85) using Thesias software. [file 1471-2350-8-1-S14.doc]

| Haplotype Effects* |  | |
| --- | --- | --- |
| AACG | - (Intercept) | |
| AGCG | OR = 1.46779 [0.96752 - 2.22672] p=0.071117 | |
| GGCG | OR = 1.04228 [0.60571 - 1.79352] p=0.881125 | |
| AACA | OR = 1.77164 [1.08265 - 2.89910] p=0.022843 | |
| GGCA | OR = 0.94765 [0.33016 - 2.72001] p=0.920387 | |
| AGCA | OR = 0.33911 [0.07821 - 1.47038] p=0.148486 | |
|  | | |
| Polymorphism 1 1/2 |  | |
| Haplotypic Background -211 | - | |
| Haplotypic Background -111 | OR = 0.71010 [0.42245 - 1.19363] p=0.196357 | |
| Haplotypic Background -112 | OR = 2.79451 [0.42941 - 18.18621] p=0.282201 | |
| Haplotypic Background -121 | - | |
|  | | |
| Polymorphism 2 2/1 |  | |
| Haplotypic Background 1-11 | OR = 1.46779 [0.96752 - 2.22672] p=0.071117 | |
| Haplotypic Background 1-12 | OR = 0.19141 [0.04223 - 0.86752] p=0.032007 | |
| Haplotypic Background 1-21 | - | |
| Haplotypic Background 2-11 | - | |
|  | | |
| Polymorphism 3 1/2 |  | |
| Haplotypic Background 12-1 | - | |
| Haplotypic Background 11-1 | - | |
| Haplotypic Background 21-1 | - | |
|  | | |
| Polymorphism 4 1/2 |  | |
| Haplotypic Background 121- | OR = 1.77164 [1.08265 - 2.89910] p=0.022843 | |
| Haplotypic Background 111- | OR = 0.23104 [0.04958 - 1.07652] p=0.062029 | |
| Haplotypic Background 211- | OR = 0.90921 [0.26037 - 3.17495] p=0.881408 | |
|  |  | |
| Haplotype frequencies | Controls (n=508) | Cases (n=98) |
| AACG | 0.291765 | 0.252653 |
| AGCG | 0.243240 | 0.295714 |
| GGCG | 0.202802 | 0.180704 |
| AACA | 0.109616 | 0.171796 |
| GGCA | 0.043847 | 0.033077 |
| AGCA | 0.046340 | 0.014515 |
| Global haplotypic effect: χ^2^ _5d.f_ =9.75, p=0.083 | | |

* Haplotypic OR by comparison to the reference with its 95% CI
