# Supplementary material for: Calpain-5 gene variants are associated with diastolic blood pressure and cholesterol levels
Source: BMC Med Genet. 2007 Jan 16;8:1. doi: 10.1186/1471-2350-8-1 (PMC1783645; doi:10.1186/1471-2350-8-1)
Supplement: Additional File 15 — T2DM. Haplotype association analysis of CAPN5 gene with Type 2 Diabetes Mellitus (T2DM) using Thesias software. [file 1471-2350-8-1-S15.doc]

| Haplotype Effects* |  | |
| --- | --- | --- |
| AACG | - (Intercept) | |
| AGCG | OR = 1.24235 [0.65170 - 2.36830] p=0.509742 | |
| GGCG | OR = 1.33312 [0.66080 - 2.68948] p=0.421989 | |
| AACA | OR = 1.45046 [0.70345 - 2.99074] p=0.313820 | |
| AGCA | OR = 2.56430 [0.89367 - 7.35802] p=0.079951 | |
| GGCA | OR = 1.09200 [0.22514 - 5.29649] p=0.913010 | |
|  | | |
| Polymorphism 1 A/G |  | |
| Haplotypic Background -GCG | OR = 1.07307 [0.51014 - 2.25719] p=0.852534 | |
| Haplotypic Background -GCA | OR = 0.42585 [0.05001 - 3.62590] p=0.434671 | |
| Haplotypic Background -GTG | - | |
| Haplotypic Background -ACG | - | |
|  | | |
| Polymorphism 2 G/A |  | |
| Haplotypic Background A-CG | OR = 0.80493 [0.42224 - 1.53445] p=0.509742 | |
| Haplotypic Background A-CA | OR = 0.56563 [0.16736 - 1.91167] p=0.359093 | |
| Haplotypic Background A-TG | - | |
| Haplotypic Background G-CG | - | |
|  | | |
| Polymorphism 3 C/T |  | |
| Haplotypic Background AG-G | - | |
| Haplotypic Background AA-G | - | |
| Haplotypic Background GG-G | - | |
|  | | |
| Polymorphism 4 G/A |  | |
| Haplotypic Background AGC- | OR = 2.06408 [0.60779 - 7.00972] p=0.245332 | |
| Haplotypic Background AAC- | OR = 1.45046 [0.70345 - 2.99074] p=0.313820 | |
| Haplotypic Background GGC- | OR = 0.81913 [0.13813 - 4.85750] p=0.826116 | |
|  |  | |
| Haplotype frequencies | Controls (n=462) | Cases (n=49) |
| AACG | 0.283906 | 0.235490 |
| AGCG | 0.264083 | 0.264316 |
| GGCG | 0.193960 | 0.203571 |
| AACA | 0.118258 | 0.139643 |
| AGCA | 0.040312 | 0.079153 |
| GGCA | 0.036236 | 0.036306 |
| Global haplotypic effect: χ^2^ _5d.f_ =3.75, p=0.586 | | |

* Haplotypic OR by comparison to the reference with its 95% CI
