# Supplementary material for: Calpain-5 gene variants are associated with diastolic blood pressure and cholesterol levels
Source: BMC Med Genet. 2007 Jan 16;8:1. doi: 10.1186/1471-2350-8-1 (PMC1783645; doi:10.1186/1471-2350-8-1)
Supplement: Additional File 16 — IFG. Haplotype association analysis of CAPN5 gene with Impaired Fasting Glucose (IFG) using Thesias software. [file 1471-2350-8-1-S16.doc]

| Haplotype Effects* |  | |
| --- | --- | --- |
| AACG | - (Intercept) | |
| AGCG | OR = 1.23003 [0.75096 - 2.01474] p=0.410864 | |
| GGCG | OR = 1.09869 [0.62008 - 1.94672] p=0.747078 | |
| AACA | OR = 1.34782 [0.72119 - 2.51891] p=0.349511 | |
| AGCA | OR = 0.81873 [0.26565 - 2.52333] p=0.727642 | |
| GGCA | OR = 1.66908 [0.57143 - 4.87520] p=0.348903 | |
|  | | |
| Polymorphism 1 A/G |  | |
| Haplotypic Background -GCG | OR = 0.89322 [0.49571 - 1.60951] p=0.707021 | |
| Haplotypic Background -GCA | OR = 2.03862 [0.37668 - 11.03326] p=0.408388 | |
| Haplotypic Background -GTG | - | |
| Haplotypic Background -ACG | - | |
|  | | |
| Polymorphism 2 G/A |  | |
| Haplotypic Background A-CG | OR = 0.81299 [0.49634 - 1.33164] p=0.410864 | |
| Haplotypic Background A-CA | OR = 1.64623 [0.46247 - 5.85998] p=0.441584 | |
| Haplotypic Background A-TG | - | |
| Haplotypic Background G-CG | - | |
|  | | |
| Polymorphism 3 C/T |  | |
| Haplotypic Background AG-G | - | |
| Haplotypic Background AA-G | - | |
| Haplotypic Background GG-G | - | |
|  | | |
| Polymorphism 4 G/A |  | |
| Haplotypic Background AGC- | OR = 0.66562 [0.19746 - 2.24367] p=0.511475 | |
| Haplotypic Background AAC- | OR = 1.34782 [0.72119 - 2.51891] p=0.349511 | |
| Haplotypic Background GGC- | OR = 1.51916 [0.43066 - 5.35888] p=0.515594 | |
|  |  | |
| Haplotype frequencies | Controls (n=488) | Cases (n=77) |
| AACG | 0.287232 | 0.220942 |
| AGCG | 0.259061 | 0.276942 |
| GGCG | 0.201237 | 0.196506 |
| AACA | 0.117201 | 0.140864 |
| GGCA | 0.032406 | 0.045227 |
| AGCA | 0.045066 | 0.034688 |
| Global haplotypic effect: χ^2^ _5d.f_ =1.96, p=0.855 | | |

* Haplotypic OR by comparison to the reference with its 95% CI
