# Supplementary material for: Calpain-5 gene variants are associated with diastolic blood pressure and cholesterol levels
Source: BMC Med Genet. 2007 Jan 16;8:1. doi: 10.1186/1471-2350-8-1 (PMC1783645; doi:10.1186/1471-2350-8-1)
Supplement: Additional File 17 — IGT. Haplotype association analysis of CAPN5 gene with Impaired Glucose Tolerance (IGT) using Thesias software. [file 1471-2350-8-1-S17.doc]

| Haplotype Effects* |  | |
| --- | --- | --- |
| AACG | - (Intercept) | |
| AGCG | OR = 0.82558 [0.48547 - 1.40397] p=0.479255 | |
| GGCG | OR = 1.15718 [0.65093 - 2.05718] p=0.618958 | |
| AACA | OR = 0.73110 [0.35400 - 1.50991] p=0.397317 | |
| AGCA | OR = 3.09970 [1.28638 - 7.46915] p=0.011695 | |
| GGCA | OR = 0.47224 [0.10411 - 2.14208] p=0.330782 | |
|  | | |
| Polymorphism 1 A/G |  | |
| Haplotypic Background -GCG | OR = 1.40165 [0.78143 - 2.51414] p=0.257350 | |
| Haplotypic Background -GCA | OR = 0.15235 [0.02302 - 1.00837] p=0.051015 | |
| Haplotypic Background -GTG | - | |
| Haplotypic Background -ACG | - | |
|  | | |
| Polymorphism 2 G/A |  | |
| Haplotypic Background A-CG | OR = 1.21126 [0.71226 - 2.05986] p=0.479255 | |
| Haplotypic Background A-CA | OR = 0.23586 [0.07819 - 0.71145] p=0.010336 | |
| Haplotypic Background A-TG | - | |
| Haplotypic Background G-CG | - | |
|  | | |
| Polymorphism 3 C/T |  | |
| Haplotypic Background AG-G | - | |
| Haplotypic Background AA-G | - | |
| Haplotypic Background GG-G | - | |
|  | | |
| Polymorphism 4 G/A |  | |
| Haplotypic Background AGC- | OR = 3.75456 [1.34356 - 10.49207] p=0.011627 | |
| Haplotypic Background AAC- | OR = 0.73110 [0.35400 - 1.50991] p=0.397317 | |
| Haplotypic Background GGC- | OR = 0.40809 [0.07895 - 2.10948] p=0.284900 | |
|  |  | |
| Haplotype frequencies | Controls (n=415) | Cases (n=74) |
| AACG | 0.276278 | 0.296953 |
| AGCG | 0.270895 | 0.236446 |
| GGCG | 0.189743 | 0.215926 |
| AACA | 0.126577 | 0.093541 |
| GGCA | 0.039749 | 0.023946 |
| AGCA | 0.031264 | 0.085216 |
| Global haplotypic effect: χ^2^ _5d.f_ =7.63, p=0.178 | | |

* Haplotypic OR by comparison to the reference with its 95% CI
