# Supplementary material for: Calpain-5 gene variants are associated with diastolic blood pressure and cholesterol levels
Source: BMC Med Genet. 2007 Jan 16;8:1. doi: 10.1186/1471-2350-8-1 (PMC1783645; doi:10.1186/1471-2350-8-1)
Supplement: Additional File 18 — SM(ATPIII). Haplotype association analysis of CAPN5 gene with Metabolic Syndrome using the ATPIII definition using Thesias software. [file 1471-2350-8-1-S18.doc]

| Haplotype Effects* |  | |
| --- | --- | --- |
| AACG |  | |
| AGCG | OR = 1.52859 [0.98103 - 2.38178] p=0.060745 | |
| GGCG | OR = 0.99422 [0.56779 - 1.74092] p=0.983813 | |
| AACA | OR = 1.80142 [1.06114 - 3.05813] p=0.029274 | |
| AGCA | OR = 1.45785 [0.57622 - 3.68842] p=0.426051 | |
| GGCA | OR = 0.94109 [0.26836 - 3.30024] p=0.924433 | |
|  | | |
| Polymorphism 1 A/G |  | |
| Haplotypic Background -GCG | OR = 0.65042 [0.36273 - 1.16627] p=0.148813 | |
| Haplotypic Background -GCA | OR = 0.64553 [0.11534 - 3.61280] p=0.618395 | |
| Haplotypic Background -GTG | - | |
| Haplotypic Background -ACG | - | |
|  | | |
| Polymorphism 2 G/A |  | |
| Haplotypic Background A-CG | OR = 0.65420 [0.41985 - 1.01934] p=0.060745 | |
| Haplotypic Background A-CA | OR = 1.23567 [0.43392 - 3.51881] p=0.691865 | |
| Haplotypic Background A-TG | - | |
| Haplotypic Background G-CG | - | |
|  | | |
| Polymorphism 3 C/T |  | |
| Haplotypic Background AG-G | - | |
| Haplotypic Background AA-G | - | |
| Haplotypic Background GG-G | - | |
|  | | |
| Polymorphism 4 G/A |  | |
| Haplotypic Background AGC- | OR = 0.95372 [0.34400 - 2.64414] p=0.927433 | |
| Haplotypic Background AAC- | OR = 1.80142 [1.06114 - 3.05813] p=0.029274 | |
| Haplotypic Background GGC- | OR = 0.94656 [0.22078 - 4.05829] p=0.941052 | |
|  | | |
| Haplotype frequencies | Controls (n=465) | Cases (n=90) |
| AACG | 0,285597 | 0,233028 |
| AGCG | 0,253288 | 0,307867 |
| GGCG | 0,205843 | 0,164272 |
| AACA | 0,111449 | 0,166244 |
| AGCA | 0,042311 | 0,049672 |
| GGCA | 0,037638 | 0,028528 |
| AGTG | 0,023114 | 0,003961 |
| Global haplotypic effect: χ^2^ _5d.f_ =7.81, p=0.167 | | |

* Haplotypic OR by comparison to the reference with its 95% CI
