# Supplementary material for: Calpain-5 gene variants are associated with diastolic blood pressure and cholesterol levels
Source: BMC Med Genet. 2007 Jan 16;8:1. doi: 10.1186/1471-2350-8-1 (PMC1783645; doi:10.1186/1471-2350-8-1)
Supplement: Additional File 19 — SM(IDF). Haplotype association analysis of CAPN5 gene with Metabolic Syndrome using the International Diabetes Federation (IDF) definition using Thesias software. [file 1471-2350-8-1-S19.doc]

| Haplotype Effects* |  | |
| --- | --- | --- |
| AACG | - (Intercept) | |
| AGCG | OR = 1.31202 [0.89235 - 1.92907] p=0.167322 | |
| GGCG | OR = 0.62816 [0.36548 - 1.07965] p=0.092442 | |
| AACA | OR = 1.37222 [0.83842 - 2.24589] p=0.208078 | |
| GGCA | OR = 1.36305 [0.59167 - 3.14010] p=0.466959 | |
| AGCA | OR = 1.09778 [0.46220 - 2.60734] p=0.832603 | |
|  | | |
| Polymorphism 1 A/G |  | |
| Haplotypic Background -GCG | OR = 0.47877 [0.27394 - 0.83675] p=0.009718 | |
| Haplotypic Background -GCA | OR = 1.24165 [0.33310 - 4.62829] p=0.747133 | |
| Haplotypic Background -GTG - | | |
| Haplotypic Background -ACG - | | |
|  | | |
| Polymorphism 2 G/A |  | |
| Haplotypic Background A-CG | OR = 0.76218 [0.51838 - 1.12064] p=0.167322 | |
| Haplotypic Background A-CA | OR = 1.25000 [0.46574 - 3.35488] p=0.657761 | |
| Haplotypic Background A-TG - | | |
| Haplotypic Background G-CG - | | |
|  | | |
| Polymorphism 3 C/T |  | |
| Haplotypic Background AG-G - | | |
| Haplotypic Background AA-G - | | |
| Haplotypic Background GG-G - | | |
|  | | |
| Polymorphism 4 G/A |  | |
| Haplotypic Background AGC- | OR = 0.83670 [0.32453 - 2.15718] p=0.712156 | |
| Haplotypic Background AAC- | OR = 1.37222 [0.83842 - 2.24589] p=0.208078 | |
| Haplotypic Background GGC- | OR = 2.16991 [0.73713 - 6.38758] p=0.159624 | |
|  |  |  |
| Haplotype frequencies | Controls (n=482) | Cases (n=119) |
| AACG | 0,284592 | 0,289744 |
| AGCG | 0,24019 | 0,294866 |
| GGCG | 0,216608 | 0,129315 |
| AACA | 0,113055 | 0,148853 |
| GGCA | 0,040121 | 0,050055 |
| AGCA | 0,040807 | 0,04479 |
| Global haplotypic effect: χ^2^ _5d.f_ =11.74, p=0.039 | | |

* Haplotypic OR by comparison to the reference with its 95% CI
